# Perspective on the pathogenic *Neisseria*: milestones, challenges, and future directions

**DOI:** 10.1038/s41541-026-01417-9

**Published:** 2026-03-13

**Authors:** Joseph A. Duncan, Ann E. Jerse, Federico Martinón-Torres, Mariagrazia Pizza, Rino Rappuoli, David S. Stephens, Christoph M. Tang

**Affiliations:** 1 Department of Medicine, Division of Infectious Diseases, University of North Carolina, Chapel Hill, USA; 2 School of Medicine, Uniformed Services University of the Health Sciences, Bethesda, MD USA; 3 Pediatrics Department, Hospital Clínico Universitario de Santiago, Santiago de Compostela, Spain; 4 Centre for Bacterial Resistance Biology (CBRB), Department of Life Sciences, Imperial, London, UK; 5 Fondazione Biotecnopolo di Siena, Siena, Italy; 6 Department of Medicine, Emory University School of Medicine, Atlanta, GA USA; 7 Sir William Dunn School of Pathology, University of Oxford, Oxford, UK

**Keywords:** Diseases, Immunology, Microbiology

## Abstract

The 24th International Pathogenic Neisseria Conference (IPNC) marked a shift toward a balanced forum addressing both meningococcal and gonococcal disease. This drove discussion on how multivalent meningococcal vaccines are enabling the WHO roadmap to defeat meningitis by 2030, while highlighting growing evidence that effective gonococcal vaccines are achievable. Major challenges remain, including antimicrobial resistance, limited genomic surveillance and incomplete understanding of pathogenesis and immune evasion. These reflections shaped this perspective piece.

## Introduction

The IPNC, which in 2025 was held in Florence (Italy), convened 316 scientists from 31 countries and 5 continents. With 214 abstracts submitted, the conference was characterized by the convergence of decades of research into translational impact. On the meningococcal side, the effort initiated in the 1960’s to develop meningococcal vaccines, culminated in the licensure of vaccines able to prevent the disease caused by all serogroups of meningococcus, enabling concrete progress toward the WHO roadmap. On the gonococcal side, since the initial attempts in the 1970’s, new evidence increasingly indicate that gonococcal disease can be prevented by vaccination.

Below we report the main topics discussed during the sessions and the round tables of the meeting.

## Evolution of the pathogenic *Neisseria*: links to clinical presentations and pathogenesis

The genus *Neisseria* is comprised of Gram-negative, oxidase positive betaproteobacteria. More than twenty-five species are found in humans and warm-blooded animals, usually identified as commensals of dental and upper respiratory mucosal surfaces^1^. The two important human pathogens of this genus, *Neisseria meningitidis* (*N. meningitidis*) and *Neisseria gonorrhoeae* (*N. gonorrhoeae*) are closely related genetically with the core housekeeping genes having ~98% nucleotide sequence identity, supporting the concept that these species likely evolved from a common unencapsulated ancestor^2^. However, since divergence, both *N. meningitidis* and *N. gonorrhoeae* have demonstrated continued genome evolution via horizontal gene transfer^3^. Linking *Neisseria* evolution, pathogenesis and epidemiology to clinical and prevention strategies continues as a major research focus of the IPNC community.

Much has been accomplished in the prevention and treatment of gonococcal and meningococcal infections, but challenges remain. For *N. meningitidis*, safe and effective meningococcal vaccines have been introduced in many countries worldwide for meningococcal disease control, including in high incidence regions such as sub-Saharan Africa. However, meningitis and/or meningococcemia outbreaks do continue to occur globally, at ~200,000 cases year, punctuated by emergence of new clonal complexes^4^. The 20% morbidity and 10-15% mortality associated with meningococcal disease remain essentially unchanged this century and antibiotic resistance including acquisition of beta-lactamases is emerging in *N. meningitidis*^5^. New clinical syndromes such as *N. meningitidis* urethritis outbreaks have also been identified^6^.

For *N. gonorrhoeae*, which primarily causes mucosal infection of the urogenital tract, rectum and oropharynx and may lead to serious complications, the progression of antibiotic resistance represents a major clinical challenge^7^, the threat of untreatable gonococcal infections is a real concern. While hope toward a partially effective gonococcal vaccine has emerged, over 80 million gonococcal cases/year are reported globally, likely representing an underestimation of the total number of cases, due to underdiagnosis. Further, *N. gonorrhoeae* “asymptomatic” mucosal infections (e.g., urogenital tract, pharynx, rectum) are underappreciated as a reservoir and in transmission and prevention strategies^8^. Infertility and perinatal complications remain significant, and the return of invasive *N. gonorrhoeae* clinical syndromes such as endocarditis is a threat. Lastly, the rapidly expanding use of C5 complement inhibitors in clinical medicine has brought the risk (2000-fold increase) for fulminant *Neisseria* infections to an increasing number of individuals^9,10^.

## Meningococcal vaccines: Where we are and where we’re headed

Meningococcal vaccines have reshaped invasive meningococcal disease (IMD) prevention worldwide. Conjugate vaccines against serogroups A, C, W, and Y have profoundly reduced IMD, in terms of septicemia and/or meningitis, through both direct protection and herd immunity. In the African meningitis belt, MenAfriVac® virtually eliminated serogroup A disease by interrupting carriage and generating herd protection^11^. In Europe, monovalent meningococcus C (MenC) conjugates (Menjugate®, Meningitec®, NeisVac-C®) produced rapid, sustained declines—near elimination in some settings—with additional herd effects (e.g., UK, Spain)^12,13^. Building on this, adolescent MenACWY programmes curb transmission: UK data show significant reductions in carriage of hyperinvasive lineages and reduction of IMD cases in all age groups, consistent with herd protection^14^.

For serogroup B, both widely licensed vaccines, 4CMenB (Bexsero®) and MenB-FHbp (rLP2086; Trumenba®), are central to direct protection strategies. Since its approval in 2013, 4CMenB has been implemented in several national immunization programmes, mainly targeting infants, and over 75 million doses have been distributed globally. Real-world evidence (RWE) for 4CMenB demonstrates meaningful effectiveness and impact across multiple countries^15–17^. The adolescent programme in South Australia achieved a ~ 70% reduction in serogroup B disease^18^. MenB-FHbp complements this toolset by targeting factor H binding protein and has robust immunogenicity and acceptable safety in adolescents and young adults^19^. Unlike conjugate vaccines, Meningococcus B (MenB) vaccines have not significantly reduced nasopharyngeal carriage, limiting indirect herd protection^15^. Nevertheless, cross-protection has emerged as an unexpected benefit. Studies in England demonstrated reductions in serogroup W disease following 4CMenB introduction^17^, while genetic similarities between *N. meningitidis* and *N. gonorrhoeae* have translated into partial protection against gonorrhoea. Observational studies reported a 33–47% reduction in gonorrhoea among 4CMenB vaccinees^16,18,20^, and the Galician Public Health Programme in Spain and the UK Joint Committee on Vaccination and Immunisation have recently recommended targeted use of 4CMenB for individuals at high risk of gonorrhoea^18,20–22^.

Looking ahead, the field is moving toward broader-spectrum meningococcal vaccines and innovative approaches. Pentavalent meningococcal conjugate–protein vaccines, MenABCWY, have been approved by the FDA for ages 10–25 years^23^. These combined formulations offer protection against serogroups A, B, C, W, Y in a single product, streamlining adolescent immunization. However, no licensed pentavalent vaccine yet exists for infants, the age group at highest risk. In parallel, Men5CV (MenFive®)—a pentavalent capsular polysaccharide conjugate vaccine—achieved WHO prequalification in 2023 to address A, C, W, Y and X in the meningitis belt, an essential step toward the 2030 roadmap^24^.

Finally, several future directions are advancing. Vector-based platforms (e.g., adenovirus) are generating durable, functional responses in preclinical meningococcal models^25^. GMMA/OMV technologies promise affordable, scalable, multivalent bacterial vaccines with intrinsic adjuvanticity^26^. Beyond classical vaccination, harnessing mucosal immunity via *Neisseria lactamica* colonization has shown reduced meningococcal carriage and cross-reactive B-cell responses in controlled human infection studies, hinting at novel ways to block acquisition and transmission^27^.

## Gonorrhoea vaccine development: status and challenges in 2025

Gonorrhoea vaccine development has made huge strides in the last 10 years, transitioning from the basic research and discovery phases of the developmental pipeline into pre-clinical testing and clinical studies. To date, the meningococcal serogroup B meningococcal 4CMenB vaccine, which is a complex outer membrane vesicle (OMV)-based vaccine, is the only vaccine that has shown evidence of protection against gonorrhoea in humans, with observational studies reporting approximately 30–40% protection^28,29^. Other OMV-based vaccine candidates include a *N. meningitidis* OMV vaccine with broader cross-reactivity against *N. gonorrhoeae* due to strategic deletion of two meningococcal outer membrane proteins^30^, gonococcal OMV formulated with microencapsulated IL-12^31,32^, GonoVac, a vaccine based on *N. gonorrhoeae* OMV native vesicles (M. Quinn et al., IPNC 2025), and a *N. gonorrhoeae* OMV expressing meningococcal PorB^33^. More recently reported OMV candidates are a combination of OMVs from genetically engineered meningococcal and gonocococcal strains^34^, meningococcal OMV expressing the gonococcal 2C7 lipopolysaccharide (P. Van der Ley et al., IPNC 2025), and *E.coli* OMV expressing six gonococcal antigens (P. Carranza et al., IPNC 2025). A transdermal, inactivated *N. gonorrhoeae* whole-cell microparticle vaccine also shows promise^35^. Promising protein subunit vaccines include a “nutritional vaccine” that targets the B subunit of the transferrin receptor^36^, and vaccines against the *N. gonorrhoeae* MetQ protein^37^ and surface-exposed loops of the *N. gonorrhoeae* MtrE protein^38^. Other progress in the field includes sophisticated new antigen-mining strategies and the availability of thousands of *N. gonorrhoeae* genomes, compared to a handful of poorly annotated sequences a decade ago, which have helped bypass the historic problem posed by *N. gonorrhoeae* antigenic variation. These resources combined with crystal structures, in silico structural predictions, and proteomic technologies have revealed conserved vaccine targets and facilitated fine-tuned mapping of promising antigens. A comprehensive protein microarray^33,39^ and multiplex ELISA for measuring the specificity of antibodies induced by 4CMenB and other complex candidate vaccines^40^ have also accelerated antigen identification.

Challenges to gonorrhea vaccine development stem from the lifestyle of the gonococcus as primarily a mucosal pathogen or an asymptomatic colonizer that does not elicit immunity to reinfection. Correlates of protection against natural infection, therefore, are not defined. Identification of correlates of vaccine-mediated protection is an active area of study, and the mechanisms of 4CMenB-mediated protection in mice appear to be multifactorial^41,42^. Serum bactericidal activity was not shown to play a protective role in 4CMenB-immunized mice, but does correlate with protection for a lipooligosaccharide peptide mimic vaccine^43^. Delineation of mechanisms by which *N. gonorrhoeae* suppresses adaptive cellular responses has prompted the inclusion of T cell analyses when examining vaccine-induced immune responses and provided a framework for novel interventions^44^. Work in this area has also informed adjuvant selection, with *N. gonorrhoeae* OMV and purified protein subunit vaccines more effective when given with Th1-inducing or Th1/Th2 balanced adjuvants than Th2-skewing adjuvants^32,45^. Gonorrhoea vaccine research is also challenged by host restrictions. To address this problem, the human urethral challenge model is being adapted for measuring vaccine efficacy against urethral infection in men^46^, and a human oropharyngeal *N. gonorrhoeae* infection model will soon be available (G. Pollock et al., IPNC 2025). Advances in animal modeling have improved the use of female mice as an in vivo system for vaccine research. Human factor H^47^ and C4B-binding protein^48^ transgenic mice provide a more rigorous test of vaccines and immunotherapies that rely on complement-mediated defenses; human transferrin (hTf) transgenic mice increase *N. gonorrhoeae* mucosal colonization, and hTf-supplemented mice support ascending infection and have been used to predict 4CMenB efficacy against pelvic inflammatory disease^49^. Transgenic mice that express human carcinoembryonic cellular adhesion molecule (hCEACAM)-5 improve *N. gonorrhoeae* cervico-vaginal infection by providing a host-restricted colonization receptor^50^, and transgenic hCEACAM-1 mice support *N. gonorrhoeae* pharyngeal colonization and have been used to test vaccine efficacy against gonococcal pharyngeal infection (J. Fegan et al., IPNC 2025). Finally, a *N. gonorrhoeae*/*Chlamydia* coinfection model is available that supports human observational data that 4CMenB efficacy against gonorrhoea is significantly reduced in individuals with a concurrent chlamydial infection^51,52^. Several research gaps and some concerns remain. The lack of clear correlates of protection continues to challenge vaccine design. The removal of the GSK vaccine, based on gonococcal OMVs, from the GSK pipeline without additional information raises questions about vaccines of similar composition, and the nature of the protective immunity. Hopefully future published analyses of the clinical and immunological data from this trial will help guide further candidate vaccines.

## Controlled human infection with *N. gonorrhoeae*, understanding pathogenesis and immunity

Because *N. gonorrhoeae* is an exclusive human pathogen that is highly adapted to life in its host, controlled human infection with *N. gonorrhoeae* has a significant role in our understanding of infections caused by this pathogen. In the 1960’s, as the principles of ethical study review and informed consent were being codified in the United States, researchers at the US Center for Disease Control conducted studies demonstrating that *N. gonorrhoeae* passaged in the laboratory for years remained capable of infecting the human male urethra^53,54^. In the 1980’s and 90’s, protocols for controlled human infection with defined dose delivery to the anterior urethra via catheter were developed by research groups at Walter Reed Army Hospital and the University of North Carolina under Institutional Review Board approved protocols^55,56^. Since their inception, these studies have only been conducted in human males due to the risk of ascending infection with potential reproductive consequences in females. Early studies focused on the natural history of gonococcal infection and utilized two lab passaged strains of *N. gonorrhoeae* (MS11 and FA1090). Both strains are infective and cause symptomatic urethritis after inoculation in the human male urethra; however, MS11 is more infective with an ID50 of ~10^3 colony-forming units (CFU) while the ID50 for FA1090 is documented to be ~10^5 CFU^57^. Subsequent studies of *N. gonorrhoeae* pathogenesis using controlled human infections utilized isogenic strains of *N. gonorrhoeae* with genetic deletion of putative virulence factors. Experiments with single strains have been used to test whether a gene or genes are required to establish infection. Experiments with two isogenic strains introduced as a competitive infection have been used to assess relative fitness during infection^57^. One such competitive infection study unexpectedly revealed that there is a biologic bottleneck in the establishment of urethral infection, with each participant developing infection with either wild-type or mutant bacteria but not both^46^. Most recently, researchers at the University of North Carolina developed a randomized controlled study that included Controlled Human Infection to assess whether 4CMenB vaccine, a licensed MenB vaccine epidemiologically tied to reduced gonococcal infections, prevented experimentally induced gonococcal urethral infection. This study is currently enrolling and remains blinded. At the time of the meeting, the overall attack rate for infection and for development of urethritis across all study participants was around 50%. This rate is significantly lower than the 90% attack rate observed in earlier studies utilizing the same strain of *N. gonorrhoeae*. While these interim findings are consistent with a protective effect of 4CMenB against urethral infection, other explanations could explain the low attack rate. One feature of vaccine testing using the controlled human infection model is the ability to correlate measured immunologic responses to outcomes of pathogen exposure, potentially revealing correlates of protection. Historically used controlled human infection models of *N. gonorrhoeae* infection have been limited to studies of infection of the human male urethra. To provide a model of extragenital *N. gonorrhoeae* infection, a pharyngeal challenge model is currently under development with plans to utilize a more recently isolated strain of *N. gonorrhoeae*. Initial studies will focus on assessing safety and identifying the inoculating dose that results in pharyngeal infection, the majority of which are expected to be asymptomatic based on clinical experience of with natural infection^58,59^. Once established this model may also prove an important tool in assessing vaccine efficacy and understanding the immunologic correlates associated with protection of this anatomic site of infection.

## Biology of plasmids in *N. gonorrhoeae*, their implications for the evolution of antimicrobial resistance and their impact on public health

The gonococcus has a long association with plasmids. The earliest available isolate of *N. gonorrhoeae* (dating from 1928) contained a small plasmid, pCryp^60^; although it is found in around 95% of isolates, the function of this plasmid remains unknown. The bacterium also harboured pConj for at least 40 years before it came to prominence in the late 1970s by conferring tetracycline resistance^61,62^. Strains harbouring p*bla*, which encodes a TEM β-lactamase, were first identified from the mid-1970s due to their high-level resistance against penicillin^63,64^. Since then, gonococcal plasmids have been rather neglected, as chromosomal mutations have emerged as the major driver of resistance against current first line agents, ceftriaxone and azithromycin^65^.

However, from a biological viewpoint, it is fascinating that a host-adapted pathogen, *N. gonorrhoeae*, contains a limited repertoire of host-adapted plasmids. pCryp is not found in other bacteria, and while pConj and p*bla* were acquired from other pathogens (*N. meningitidis* and *Haemophilus ducreyi*, respectively), they are largely restricted to the gonococcus and confer no obvious fitness costs^62,66–68^. Illustrating this, pConj is stably inherited over 100 generations, even in the absence of selective pressure, and did not disappear after tetracycline was discontinued for treating gonorrhoea^67^. This contrasts with the dynamics of many resistance plasmids in other bacteria which impose a measurable burden on growth or competitiveness. The adaptation of plasmids to the gonococcus raises fundamental questions about the interaction between the host and these mobile genetic elements.

pConj is not only notable for its stability but is also one of the most mobile naturally occurring plasmids described. In the lab, pConj can be transferred by conjugation into nearly 100% of recipients in a standard mating between isogenic strains^62^. Despite this, pConj is unevenly distributed in the gonococcal population. The plasmid is associated with particular lineages and entirely absent from others^69^. The reasons for this patchy distribution remain unclear, although the prevalence of pConj encoding tetracycline resistance (mediated by tetM) and p*bla* is highest in low- and middle-income countries^69^.

This simple observation has substantial public health implications. Because pConj can confer high-level tetracycline/doxycycline resistance, its presence in a lineage effectively nullifies the use of these antibiotics. This means that doxycycline post-exposure prophylaxis (DoxyPEP), a population-level strategy for preventing sexually transmitted infection, is undermined wherever isolates containing pConj circulate^70,71^. Given the virtually ubiquitous presence of pConj in isolates from coastal Kenya^72^, it was no surprise that DoxyPEP was ineffective at reducing gonococcal infection^73^. Therefore, clarifying the molecular determinants of pConj spread/acquisition and maintenance is a research priority.

p*bla*, in contrast, illustrates the ongoing evolutionary trajectory of plasmid-borne resistance in the gonococcus. Molecular and phylogenetic evidence indicates that the plasmid has diversified within *N. gonorrhoeae*. Three main p*bla* variants now circulate globally, often associated with particular gonococcal lineages^74^. p*bla*.2 (the largest variant) inflicts a considerable burden on the gonococcus and appears to be on the wane^74,75^. Instead, by lacking redundant genes involved in replication^76^, p*bla*.1 and p*bla*.3 are carried without cost and seem to be on the increase^74^. This mirrors the evolution of bacterial pathogens to a single host, which is often marked by gene loss. Crucially, p*bla* variants encoding a TEM-135 β-lactamase exhibit higher resistance and are also just one mutation away from becoming an extended-spectrum β-lactamase (ESBL)^74^. The emergence of an ESBL plasmid in the gonococcus would render first-line cephalosporins ineffective. In this respect, p*bla* represents an evolutionary threat that requires close monitoring, particularly where TEM-135 expressing p*bla* is found.

An additional unusual feature of gonococcal plasmid biology is the interplay between p*bla* and pConj^74^. The dissemination of p*bla* within the gonococcal population appears to be promoted by the presence of pConj; the conjugative plasmid can facilitate the transfer of p*bla*, with the two plasmids frequently co-occurring^69^. This creates a partnership in which selection for pConj will also select for p*bla*. As a result, the risk of p*bla* becoming an ESBL plasmid will be magnified by any intervention (such as DoxyPEP) that selects for the spread of pConj and isolates carrying it.

Despite these insights, several fundamental questions remain unanswered. Why do certain gonococcal lineages carry plasmids while others seem resistant? How do interactions between plasmids, such as the co-occurrence of pConj and p*bla*, shape their long-term evolution? How likely is it that p*bla* will evolve into an ESBL, and under what conditions? And critically, how will the widespread implementation of DoxyPEP influence the evolution of plasmid-encoded resistance in the gonococcus? Recent evidence of a rise in pConj-carrying isolates was predictable and makes the outcome clear^77^.

With Doxycicline resistance increasing, it is likely that DoxyPEP will become less effective against gonococcal infection, given that as pConj has no fitness costs and is highly mobile. The stability of the plasmid also means there will be no going back. The partnership between pConj and p*bla* means that selection for the former might also promote the spread of p*bla*, which has the potential to evolve into an ESBL plasmid. Therefore, enhanced surveillance of gonococcal plasmids is needed in the face of Doxypep, a partially protective public health intervention.

## Defeating meningitis by 2030. A global road map

The recent licensure of the pentavalent conjugate vaccine to immunize against meningococcal serogroups ACYWX provided the opportunity to organize two round tables dedicated to the concrete possibility to eliminate meningococcal meningitis from the African continent.

The round tables, organized by Marie Pierre Preziosi, focused on the WHO roadmap to defeat meningitis by 2030^78^ and how this could be implemented in the African meningitis belt. The vision of the WHO roadmap is a world free of meningitis, which can be achieved by eliminating meningitis epidemics, by reducing the vaccine-preventable cases, by reducing the disability and improving the quality of life after meningitis. The strategy is based on five pillars, which are prevention and epidemic control, diagnosis and treatment, disease surveillance, support and care for people affected by meningitis and advocacy and engagement. The discussion, which saw the participation of several African scientists, touched most of the pillars but focused mainly on the successful experience of the conjugate vaccine against meningococcus A (MenAfrivac®) and how this could guide the implementation of the pentavalent vaccine. The conclusion was that, given that MenAfrivac® had completely eliminated the disease caused by serogroup A from the countries that implemented the vaccination, the pentavalent vaccine provides a unique opportunity to meet the goal of the WHO roadmap by eliminating meningococcal meningitis from Africa by 2030. However, in order to achieve the goal, we need to make sure that all countries have access to affordable vaccines, that appropriate implementation plans are in place to secure high coverage, and that disease surveillance will be in place.

## Conclusions

**Fig. 1:**
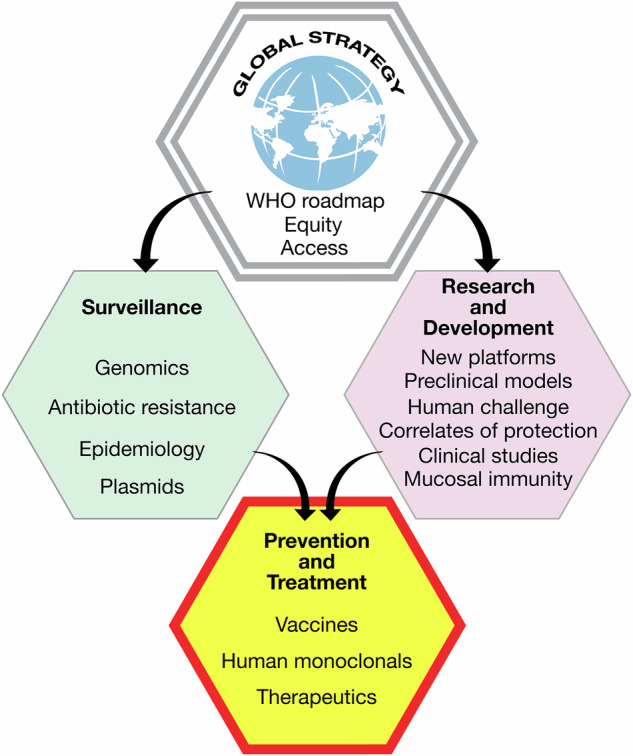
Priorities identified during the IPNC for the future of the pathogenic *Neisseria* field.

The priorities identified during the IPNC for the future of the pathogenic *Neisseria* field and their alignment with global public health strategy are illustrated in Fig. 1.

The WHO Defeating Meningitis by 2030 roadmap is a landmark global health policy that aims to eliminate meningitis as a public health threat through vaccination, surveillance, rapid diagnosis, and care for those affected. Vaccines that provide broad protection against pathogenic strains across all ages and regions are now available, enabling countries to make significant progress toward this goal.

Alongside preventive measures, continued development of novel therapeutics and monoclonal antibodies (mAbs) is essential to improve treatment outcomes, address antimicrobial resistance, and provide targeted interventions for severe infections, complementing vaccination strategies and strengthening overall disease control. However, challenges remain: meningococcal and gonococcal infections remain highly clinically relevant on a global scale in 2025, and both *N. meningitidis* and *N. gonorrhoeae* continue to evolve and adapt as human pathogens, emphasizing the need for ongoing global laboratory-based surveillance. Surveillance strategies must also account for genetic mechanisms that influence transmission and resistance: plasmids in *N. gonorrhoeae* are not just curiosities, they are stable, highly adapted genetic elements that directly undermine current and emerging strategies for controlling gonorrhoea, making their monitoring vital for anticipating epidemiological trends and informing public health planning. Population genomics is an increasingly important approach to identifying, confirming and understanding the evolution of *N. gonorrhoeae* and *N. meningitidis* determinants of pathogenesis and for selecting vaccine, mAbs and therapeutic targets, ensuring preventive and therapeutic strategies remain effective over time. Parallel advances in gonorrhoea research have been reinvigorated by promising studies in humans, and by new models to measure immune responses, supporting integrated vaccine and therapeutic development. Controlled human studies are not only reshaping our understanding of *N. gonorrhoeae* pathogenesis but also opening the way to the discovery of correlates of protection, which are essential for vaccine licensure and policy decisions. Ultimately, defeating meningitis and controlling gonorrhoea will require sustained international collaboration: the IPNC continues to provide a unique forum for scientists to share data, coordinate strategies, and shape research agendas, and only through continued effort, coordination, and investment will it be possible to eliminate *Neisseria* species as public health threats.
